# Regional gap and sustainable development of interpreting level in mainland China: A statistics and GIS-based study

**DOI:** 10.1371/journal.pone.0295505

**Published:** 2024-03-29

**Authors:** Tianyuan Xu, Ling Xue, Hengxing Xiang

**Affiliations:** 1 School of Foreign Studies, Zhongnan University of Economics and Law, Wuhan, Hubei, China; 2 State Key Laboratory of Black Soils Conservation and Utilization, Northeast Institute of Geography and Agroecology, Chinese Academy of Sciences, Changchun, Jilin, China; West Pomeranian University of Technology, POLAND

## Abstract

Against the backdrop of globalization, interpreting, a translation communicative activity in a verbal way, plays an increasingly important role in international communications and exchanges. In response to this world pattern, the Chinese government attaches great importance to the interpreting industry. However, due to the national condition of uneven regional development, the English interpreting level across China is also unbalanced. Confronting this circumstance, previous research only stagnates at the level of recognizing the problem, but very few studies have attempted to solve the problem. Thus, the current study aims to figure out the regional interpreting level in mainland China by establishing and utilizing an innovative indicator system based on statistics and geography technologies. Based on the literature review and empirical questionnaire survey from different stakeholders, the study proposes an indicator system containing 3 first-level factors and 7 second-level factors to measure regional English interpreting levels. The weight of each indicator and scoring method is laid down based on factor analysis and interval marking. In addition, putting the innovative indicator system into practice, a total of 38 groups of regional data are collected to rank the regional interpreting level across China. Integrating with GIS and statistical techniques, the result visually shows that the English interpreting level across China is uneven at present: higher in the southern and eastern parts of China compared to that of northern and western China, which is unfriendly to sustainable development in the future. Facing this reality, a following-up analysis has been made for offering explanations of the results and suggestions for regional interpreting sustainable development.

## 1. Introduction

Over the past two centuries, globalization all along has been a buzzword and the theme of the times [1, 2]. Against this backdrop, communications and exchanges among countries across the world have witnessed a great expansion [3]. Interpreting, a translation communicative activity in a verbal way, has naturally carved a place in the context of international interactions for its promptness and effectiveness [4].

In response to the globalizing world pattern, the Chinese government attaches great importance to the interpreting industry over the past few decades. Accordingly, the bloom of the English interpreting industry in China has been outstanding, which can be embodied in three major aspects, interpreting qualification test, interpreting training on and off campus [5–8]. Firstly, various interpreting qualification tests such as Shanghai Interpretation Accreditation (SIA), China Accreditation Test for Translators and Interpreters (CATTI) and English Interpreting Certificate (EIC) were designed to assess the interpreting competence of would-be and professional interpreters in a fair manner [7, 9]. Thereinto, CATTI is now recognized as the most authoritative translation and interpreting competence qualification accreditation test in China due to its great performance in reliability and validity [10]. Secondly, apart from multitudinous interpreting tests, interpreting training strongly supported by the Chinese government can also reflect the flourishing interpreting industry in China. Since the first 15 Master of Translation and Interpreting (MTI) pilot university training programs ratified by the Ministry of Education of the People’s Republic of China in 2007, the number of campus training institutions including universities and colleges has surged to 215 by the end of 2016 [11]. Lastly, not limited to interpreting training on campus, many institutions off campus in China also provide interpreting services for various occasions and interpreting training courses for potential interpreting talents to meet the market demands [12]. Thus, the rapid development of the interpreting industry in China cannot achieve without the joint effort of holding related qualification tests, carrying out interpreting training on and off campus.

However, due to China’s regional inequality in many aspects such as economy, education and innovation capacity, it is unpractical to attain balanced development in the field of interpreting currently in different parts of China [13–15]. Confronting the existing circumstances, prior research only stagnates at the level of recognizing the problem, but very few studies have attempted to solve the problem. Therefore, the current study aims to figure out the regional interpreting level in mainland China by establishing and utilizing an innovative indicator system based on statistics and geography technologies. In general, The study can be roughly divided into two steps: (1) building an indicator system assessing the regional interpreting level; (2) inputting a total of 38 groups of regional data into the innovative indicator system to score and rank the regional interpreting level in China.

## 2. Method

### 2.1 Area of study

**Fig 1:**
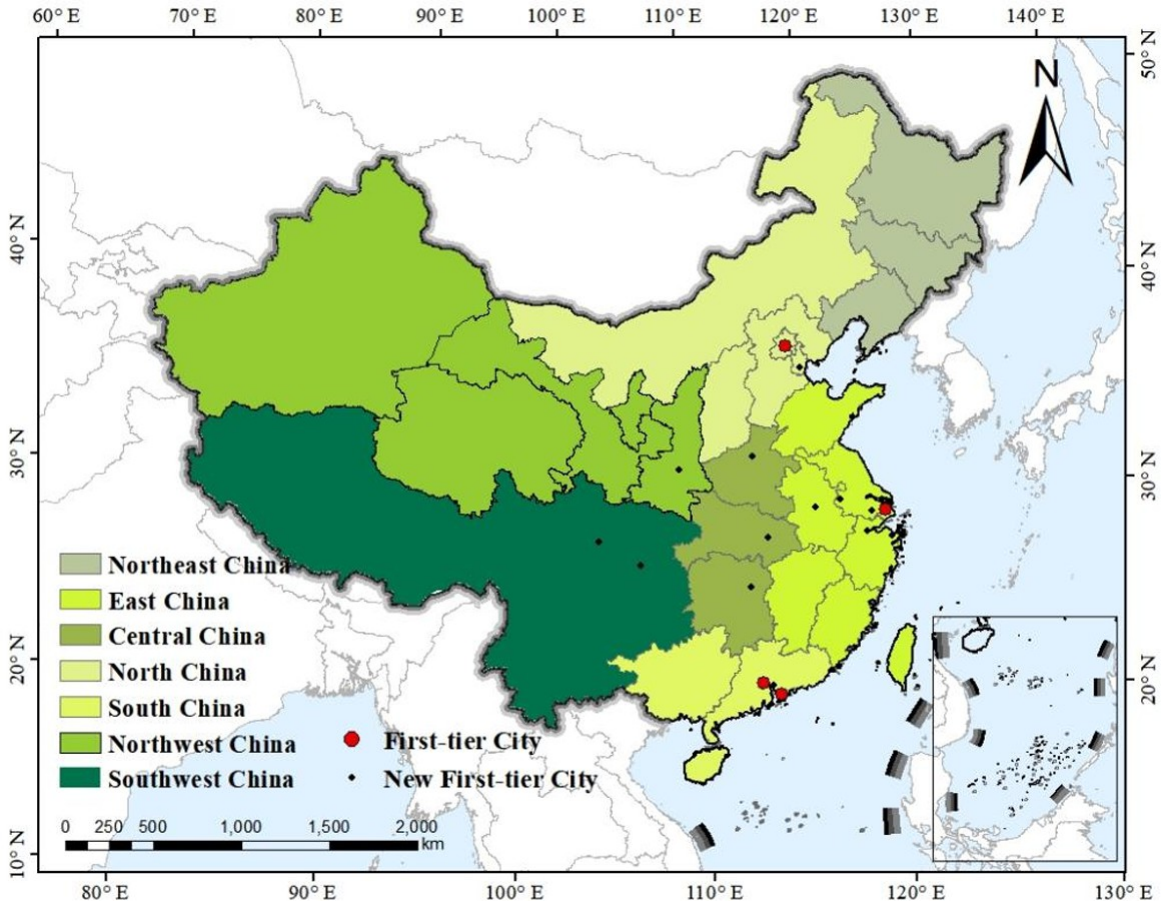
Geographical map of China.

China is located in the east of Asia and on the west coast of the Pacific Ocean. It is the third largest country in the world, covering an area of approximately 9.6 million km^2^ and spanning five geographical time zones. There are 34 provincial-level administrative regions in China, including 23 provinces, 5 autonomous regions, 4 municipalities directly under the central government and 2 special administrative regions [16]. According to the geographical feature, China consists of seven geographical divisions, namely North China, East China, Central China, South China, Northeast China, Southwest China and Northwest China. Of the four first-tier cities (standing for the most developed areas of the country) in China, one is located in north China (Beijing), one in east China (shanghai) and two in south China (Guangzhou and Shenzhen). With regard to new first-tier cities ranked by CBNweekly in 2022, 15 cities are selected on the list based on five major indicators, concentration degree of commercial resources, city pivotability, population activeness, lifestyle diversity and future plasticity [17]. Spotlighting the regional distribution of these new first-tier cities 15 cities, six are located in East China (Hangzhou, Ningbo, Nanjing, Suzhou, Hefei and Qingdao), three in Central China (Wuhan, Zhengzhou and Changsha), two in Southwest China (Chengdu and Chongqing), two in South China (Dongguan and Foshan), one in Northwest China (Xi’an) and one in North China (Tianjin). The geographical distribution map of China is shown as Fig 1 below. Four big red dots stand for China’s first-tier cities, and little black dots represent 15 China’s new first-tier cities.

The present paper is a regional study focusing on the geographical scope of mainland China. The result of the regional English interpreting level will be presented in both the geographical-level administrative regions and provincial-level administrative regions. Related figures will be shown in the result section.

### 2.2 Construction of indicator system

**Fig 2:**
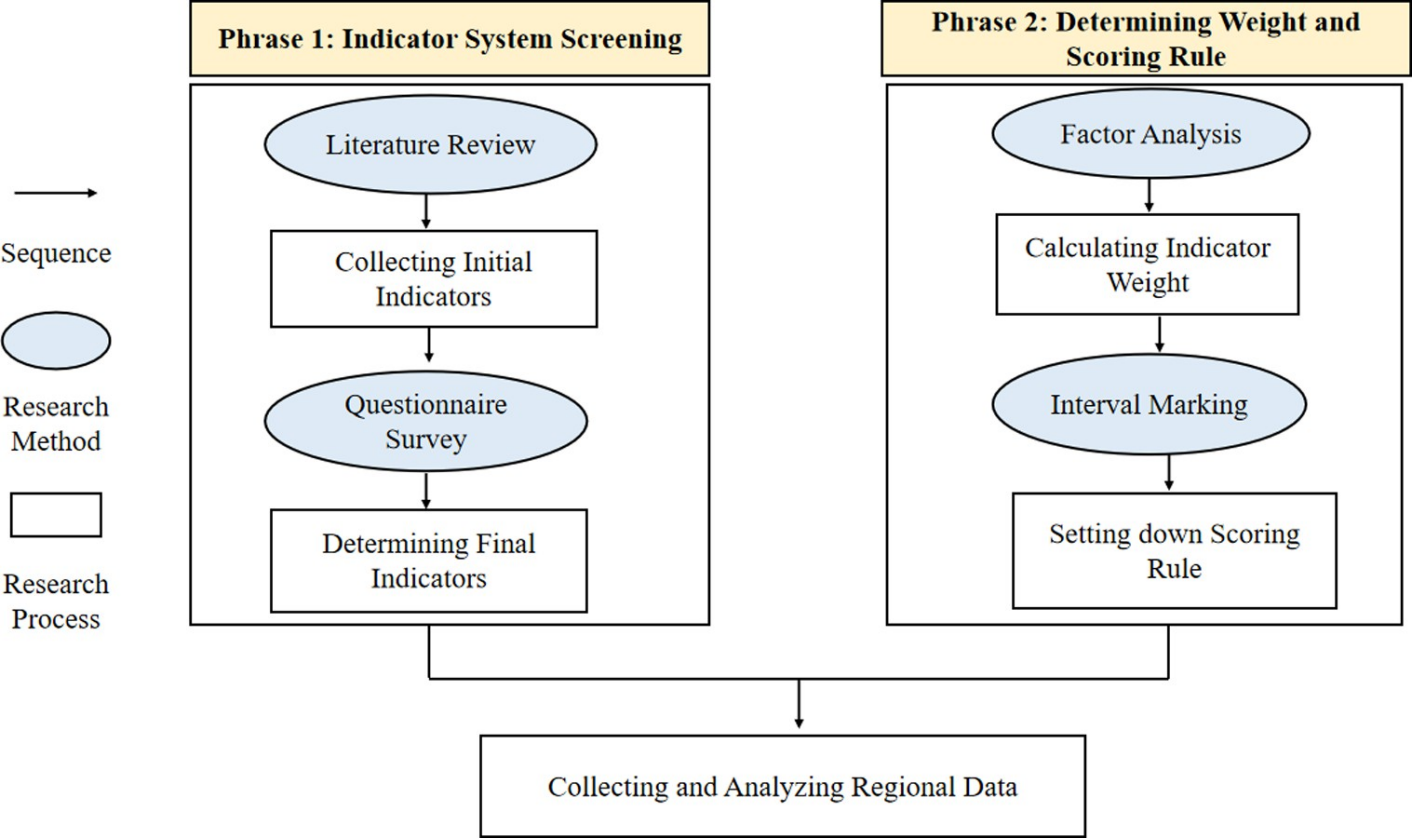
The method adopted in this study.

To construct an objective and scientific indicator system to evaluate the regional development level of English interpreting, both qualitative and quantitative analysis are applied in this research. Shown as Fig 2, the construction process can be roughly divided into two major phases: indicator system screening and determining weight and scoring rule. In the first phase, the qualitative method, literature review, is mainly utilized to define and collect initial indicators to establish an indicator pool, and questionnaire-based quantitative analysis is applied for determining the final indicator. In the second phase, quantitative methods including factor analysis and interval marking are combined for computing indicator weight and setting down scoring rules. Fig 2 below shows the method adopted in this study.

#### 2.2.1 Conceptualizing the regional level of interpreting

Though a solid foundation has been laid on testing and assessing aptitude for both practitioners and trainee interpreters, with evidence-based research adopting a wide range of paradigms and perspectives [18], empirical studies measuring interpreting performance at the regional level are still under-provided. Yet from the previous studies, valuable references can be drawn and benchmarks can thus be made. Reviewing works of interpreting studies from 2004–2009, Liu Minhua found two outstanding methodological approaches, namely quantitative and qualitative, and suggested that with the popularity of ‘mixed method research’, it is increasingly difficult to draw a line between quantitative and qualitative studies and data should be collected and analyzed both quantitatively and qualitatively [19]. Inspired by the research trend and determined to open itself to wider international academic purview, this study adopts the scale (quantity) and standard (quality) research method in evaluating the regional level of English interpreting.

#### 2.2.2 Collecting and determining indicators

Now that the definition of regional English interpreting level is explicit, collecting indicators that could reflect the theme is the next step to constructing the indicator system. It is worth mentioning before coming into operation that the principle of indicator selection should follow the rule of hierarchy, simplicity, comprehensiveness and operability [20]. To establish an evaluation system that conformed to the above principles, the literature review is applied to assemble all potential indexes to provide selections for further screening afterward [7, 9, 21–23]. Taking all stakeholders into consideration (interpreting discipline experts, professional interpreters and student interpreters), the indicator system can be generally divided into three major categories: interpreting test, level of interpreting on campus as well as level of interpreting off campus.

*A*. *Interpreting tests*. At the mere mention of assessing the level of a certain skill, it is inescapable to be associated with relevant skill tests. For example, students from non-native English-speaking countries who plan to complete a degree in an English-speaking country are required to attend English competence tests such as IELTS (International English Language Testing System) or TOEFL (Test of English as a Foreign Language), which is one of the best approaches for the applied school to directly perceived the candidate’s English level [24]. Similarly, English interpreting tests can also reflect English interpreting levels to a very large extent. Hence, the quantity and quality of English interpreting tests in different regions can reflect the level of regional English interpreting level in China from one aspect. Chinese scholars including Xiaolu Yuan, Ming Huang and Junping Liu have focused on China’s interpreting accreditation exam and overviewed the situation of interpreting tests in China. As early as 1995, Shanghai, for the first time, rolled out the interpreting qualification certificate test, effectively promoting the development of interpreting teaching and testing. It has taken the first step on the road of standardization of the interpreting talent market [25]. Since then, more than ten types of interpreting tests sprang up all over China in the following several decades [7]. Of these diverse interpreting tests, three of them have won universal trust from all sectors of society, China Accreditation Test for Translators and Interpreters (CATTI), Shanghai Interpretation Accreditation and English Interpreting Certificate (EIC). Thereinto, CATTI focuses most people’s attention upon and is generally acknowledged as the most authoritative translation and interpretation proficiency accreditation test in China [25, 26]. It is a state-level vocational test entrusted by the Ministry of Human Resources and Social Security (MHRSS) of the People’s Republic of China, with test center covering every province, autonomous region and municipality in China [26]. The other two interpreting tests, Shanghai Interpretation Accreditation and English Interpreting Certificate, are both sponsored by the Chinese top-ranked university, Shanghai International Studies University and Xiamen University separately. Unlike the full coverage of the CATTI testing center, the rest two interpreting tests only host exams in some areas. Facing the disparate penetration degree in different regions, the number of interpreting test types can be counted up to mirror the quantity of regional English interpreting test levels. As for assessing its quality, the passing rate of the interpreting test can be utilized to reach the target.

*B*. *Level of interpreting on campus*. Witnessing the prosperous development of interpreting tests, many educational specialists have put forward an idea of linking interpreting tests and Chinese MTI (Master of Translation and Interpreting) education to cultivate more applied talents [9, 27]. A growing number of interpreting teachers agree with this view with an increasing number of universities and colleges setting up translation and interpreting major. Therefore, the level of interpreting on campus (level of MTI) is designed as another indicator for evaluating English interpreting level, and both quantity and quality should be taken into account as before. Firstly established in 2007, MTI has been approved by the Academic Degrees Committee of The State Council, becoming one of the professional degrees in China. Over the past 15 years, the number of colleges and universities authorized to set up MTI has shot up from the initial 15 to the current 316. These colleges and universities distribute throughout the whole country, thus the number of them can embody the quantity of interpreting levels on campus. Regarding the quality of the interpreting level on campus, it has been studied by Research Centre for Country-specific Translation and Interpretation Capacity (RECTIC) attached to Beijing Foreign Studies University. RECTIC put forward an index system of translation and interpreting ability for Chinese colleges and universities in 2021 [28]. Since the index system integrates translation and interpreting dimensions together, it is unsuited to indiscriminately copy from it to the present study. Consequently, the indicator selected in this research takes the previous index system as a reference, and is designed to measure the MTI level from two perspectives, teachers’ scientific research level and students’ performance in the national interpreting contest.

**Fig 3:**
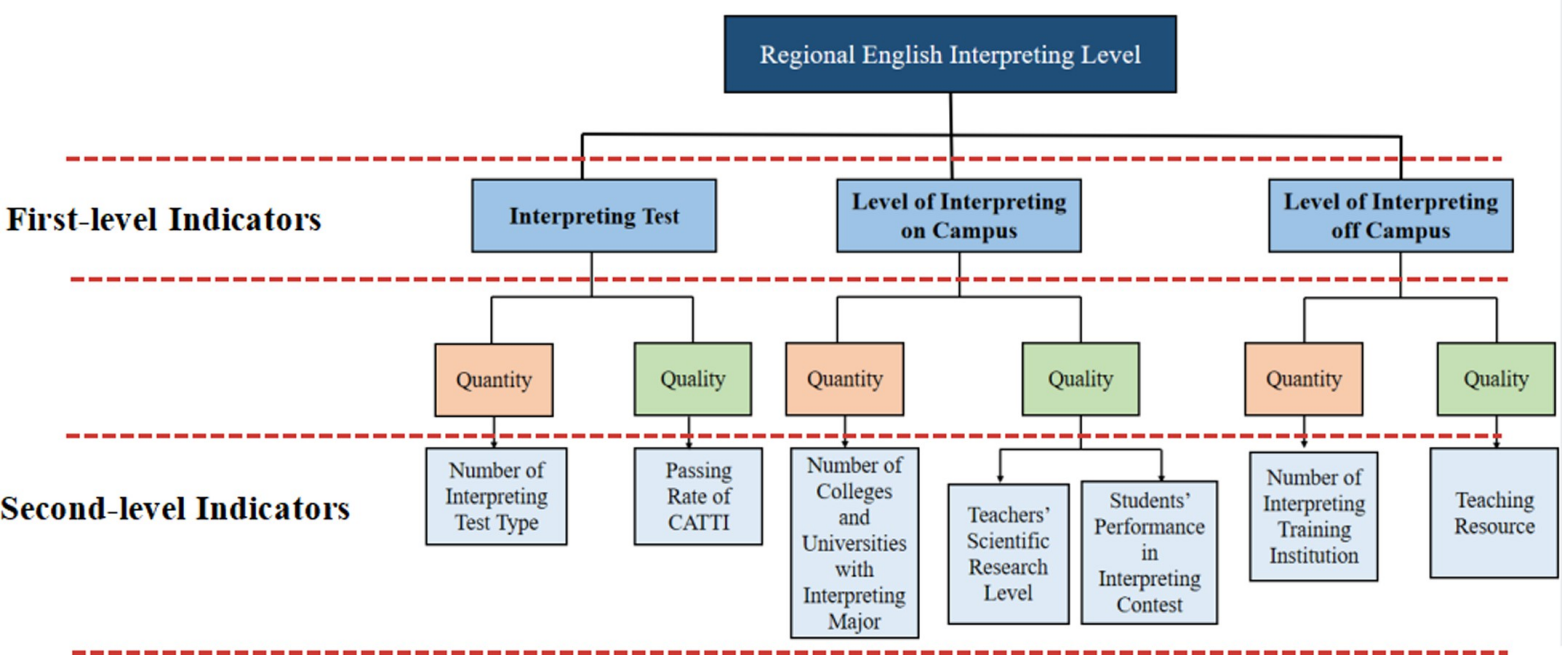
Initial indicators collected based on literature review.

*C*. *Level of interpreting off campus*. Only measuring the regional level of interpreting tests and MTI is far from enough. The level of interpreting off campus is also of great significance, which can represent the situation of the interpreting market. As the demand for international communication and exchange has increased with the accelerating pace of globalization, interpreting is needed for more kinds of occasions. Given this phenomenon, many agencies in China have started to offer interpreting services and courses to serve the interpreting free market. These interpreting institutions pay more attention to interpreting practice, such as registering as interpreting service providers for international agencies including United Nations, and cultivating interpreting trainees into professional interpreters via courses and internships. The addition of interpreting training courses also has enriched the English training market, helping interpreting amateurs receive professional training [29]. Due to the property of marketization, unlike setting up MTI courses in colleges and universities all over China, these interpreting agencies are market-oriented, thus not existing in every region. As a consequence, the quantity and quality of these off-campus interpreting agencies can be regarded as a reflection of the regional English interpreting level. To assess these two aspects, the number of interpreting agencies and teaching resource data are collected. To sum up, based on the literature review, three first-level indicators and seven second-level indicators are collected (shown as Fig 3).

To further determine final indicators, a questionnaire survey (attached in S1 Appendix) is applied to get feedback from different stakeholders, including 10 interpreting discipline experts, 10 professional interpreters and 10 student interpreters, who all possess abundant knowledge reserve about interpreting. The questionnaire is designed in the Likert scale, one of the most widely accepted scales in survey research. By choosing the score from 1 to 5, participants could express their level of agreement with the statement (e.g. I agree that the number of interpreting test types in different regions can represent the local interpreting level to some degree. Choosing 1 for disagree strongly, 2 disagree a little, 3 neither agree nor disagree, 4 agree a little and 5 agree strongly). An open-ended question is added at the end of the questionnaire to ask subjects whether there are some other indicators that can be put into the system from their perspectives. After finalizing the indicator system evaluating regional English interpreting level, the next step is to validate the weight of each indicator and its scoring method.

#### 2.2.3 Validating indicator weight and scoring method

The determination of indicator weight and setting scoring standards is an essential segment of the whole evaluation system. To achieve it, factor analysis and interval marking methods are applied through the software SPSS (Statistical Product Service Solutions) and EXCEL.

Factor analysis is one of the most common and acknowledged statistical techniques to extract common factors from variable groups. Precisely because of its essence, it can be used to calculate indicator weight based on information concentration. The procedure of applying factor analysis to validate weight is as follows [30, 31]:

1. Testing whether factor analysis is suitable for present statistics by KMO and Bartlett’s Test.
2. Obtaining the variance contribution rate Pi and component score coefficient βi by factor analysis on each indicator.
3. Calculating weight for each indicator by formula (1≤j≤n,1≤k≤y, y represents the number of common factors extracted by factor analysis).

Following weight fixing, setting down the scoring rule is the last step of constructing the complete indicator system. The interval marking method, a highly workable method, is applied to convert raw data into scores with a unified standard to rank the interpreting level of different regions in the result section. After setting intervals and their corresponding marks in line with the case, the score of each index deserves is then determined according to which interval the original data falls in. Up to this point, the indicator system has been fully built, and the rest steps leave to collecting and analyzing regional data.

### 2.3 Collecting and analyzing regional data

Following the principle of timeliness, correctness and reliability, data for the year 2021 is collected across the different regions of China (except the data in Hong Kong, Taiwan and Macao). To ensure dependability, data for most indicators are gathered from relevant official websites such as the number of interpreting test types, the passing rate of CATTI, the number of colleges and universities with interpreting major, students’ awards situation of interpreting contests and the number of interpreting training institutions. As for data on the other two indicators, it is collected according to quantifiable correlative. To be specific, teachers’ scientific research level is assessed by the number of paper they have published on authoritative and influential academic journals included in SCI (Science Citation Index), SSCI (Social Sciences Citation Index), CSSCI (Chinese Social Sciences Citation Index) and PKU (Core journal of Peking University). Similarly, the quality of teaching resources in interpreting institutions is measured by the number of interpreting teachers with the identity of AIIC (International Association of Conference Interpreters, the only global professional association for conference interpenetrating) member.

Apart from conventional data processing software EXCEL, a professional computer mapping software, ArcGIS, is also applied into use to analyze study data. It is worth noting that all the geographic data input in ArcGIS are acquired from a publicly available website (https://www.resdc.cn/Default.aspx). The combination of ArcGIS helps visualize the regional situation across the country. The result of the present study will be illustrated in the next section.

## 3. Results

### 3.1 Finalized version of the indicator system

On the basis of a literature review and questionnaire survey to different stakeholders, indicators are finalized. The indicators selected after conducting the questionnaire survey are only fine-tuned based on the initial ones. For example, the passing rate of CATTI is refined to the passing rate of CATTI Level 1, CATTI Level 2 and CATTI Level 3 interpreting test (level 1 represents the level of senior interpreter, level 2 for interpreter and level 3 for assistant interpreter).

In the process of calculating weight, KMO and Bartlett’s Test is the first step to examine whether factor analysis is suitable in the present study. Shown as Table 1, the KMO (Kaiser-Meyer Olkin) coefficient is 0.749, representing the current data is suitable for conducting factor analysis [32].

**Table 1:** KMO and Bartlett’s test.

KMO and Bartlett’s Test		
Kaiser-Meyer-Olkin Measure of Sampling Adequacy.		0.749
Bartlett’s Test of Sphericity	Approx. Chi-Square	113.616
	df	36
	Sig.	< .001

Outputting the result of factor analysis through SPSS, variance contribution rate and component score coefficient are shown as Tables 2 and 3 below. Three principal factors are extracted.

**Table 2:** Eigenvalues and variance contribution rate.

Component	Initial Eigenvalues			Extraction Sums of Squared Loadings			Rotation Sums of Squared Loadings		
	Total	% of Variance	Cumulative %	Total	% of Variance	Cumulative %	Total	% of Variance	Cumulative %
1	3.838	42.646	42.646	3.838	42.646	42.646	3.521	39.125	39.125
2	1.313	14.585	57.231	1.313	14.585	57.231	1.571	17.457	56.582
3	1.062	11.795	69.026	1.062	11.795	69.026	1.12	12.445	69.026
4	0.971	10.79	79.816						
5	0.67	7.45	87.266						
6	0.474	5.267	92.532						
7	0.38	4.228	96.76						
8	0.169	1.878	98.638						
9	0.123	1.362	100						

**Table 3:** Component and rotated component matrixa.

Indicator	Component Matrixa			Rotated Component Matrixa		
	Component			Component		
	1	2	3	1	2	3
Number of Interpreting Test Type	0.286	-0.628	0.027	0.063	0.628	-0.282
CATTI Level-1 Interpreting Test	0.393	0.432	-0.697	0.607	-0.532	-0.418
CATTI Level-2 Interpreting Test	0.008	0.634	0.671	0.113	-0.214	0.891
CATTI Level-3 Interpreting Test	0.564	-0.366	0.238	0.377	0.605	0.021
Number of Colleges and Universities with Interpreting Major	0.49	-0.361	0.127	0.325	0.525	-0.072
Teachers’ Scientific Research Level	0.789	0.028	-0.184	0.774	0.17	-0.169
Students’ Performance in Interpreting Contest	0.922	0.064	0.089	0.871	0.311	0.085
Number of Interpreting Training Institution	0.911	0.19	0.056	0.905	0.189	0.116
Teaching Resource	0.86	0.156	0.09	0.843	0.214	0.131

Based on the formula mentioned above, the specific weight of each indicator is calculated, and the whole indicator system is finalized. The indicator system and its weight are presented in Table 4. The weight of the first-level indicator interpreting test, level of interpreting on campus and level of interpreting off campus are 0.4155, 0.3309 and 0.2536 respectively. Further speaking, the weight of four second-level indicators of interpreting tests are 0.0775, 0.1447, 0.1026 and 0.0907, representing the proportion of the number of interpreting test types and CATTI level-1, 2 and 3 interpreting tests. In terms of the second-level indicator of the level of interpreting on campus, the weight of the number of colleges and universities with interpreting major, teachers’ scientific research level and students’ performance in interpreting contests are 0.0829, 0.1166 and 0.1314 respectively. The weight of the last two second-level indicators of the level of interpreting off campus, the number of interpreting training institutions and teaching resources, are 0.1288 and 0.1247 in the order given.

**Table 4:** The indicator system and its weight.

First-level Indicator	Weight	Second-level Indicator	Weight
Interpreting Test	0.4155	Number of Interpreting Test Type	0.0775
		CATTI Level-1 Interpreting Test	0.1447
		CATTI Level-2 Interpreting Test	0.1026
		CATTI Level-3 Interpreting Test	0.0907
Level of Interpreting on Campus	0.3309	Number of Colleges and Universities with Interpreting Major	0.0829
		Teachers’ Scientific Research Level	0.1166
		Students’ Performance in Interpreting Contest	0.1314
Level of Interpreting off Campus	0.2536	Number of Interpreting Training Institution	0.1288
		Teaching Resource	0.1247

### 3.2 Results on English interpreting level in different geographical-level administrative regions

**Fig 4:**
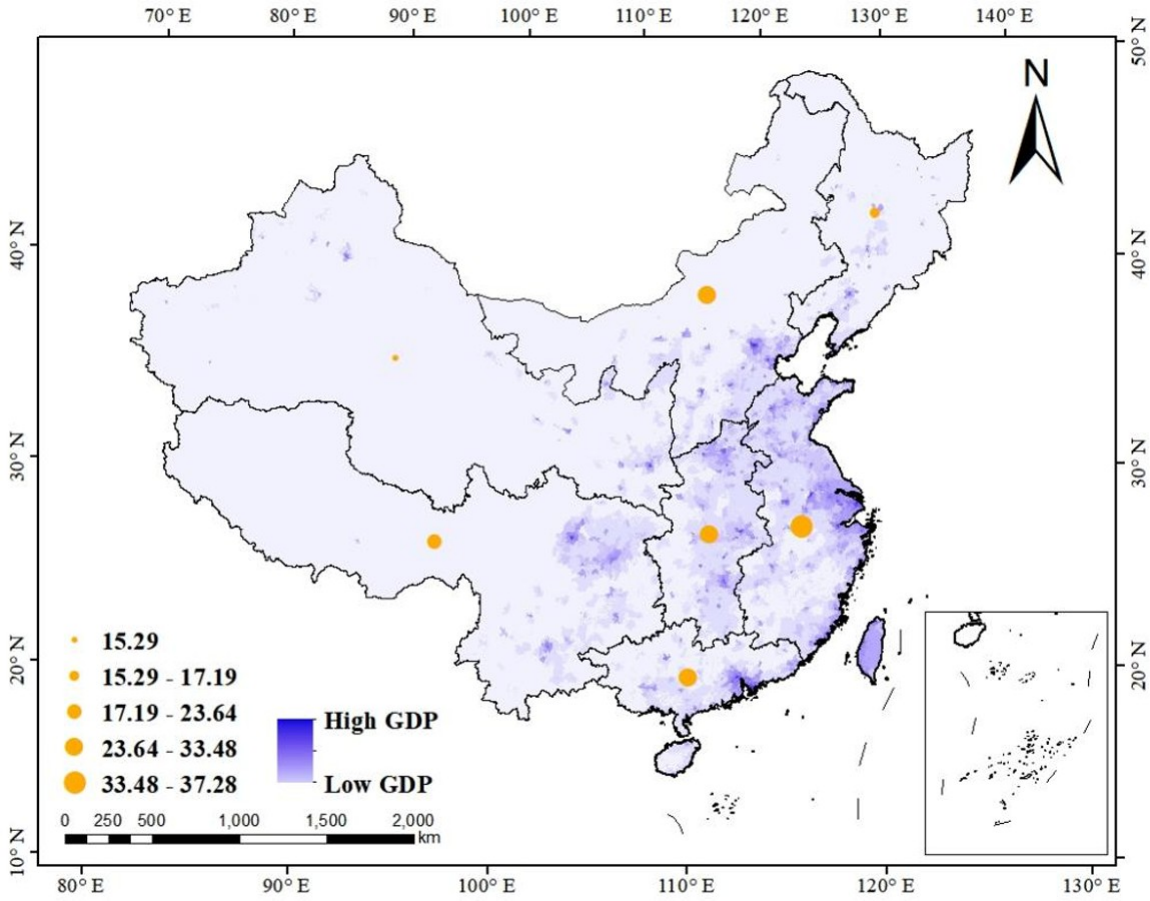
The overall English interpreting level in different geographical-level administrative region.

Calculating the mark of each area according to weight and scoring rule, a list of regional scores is generated (shown as Table 5). According to geographical division, China consists of seven regions, namely North China, East China, Central China, South China, Northeast China, Southwest China and Northwest China. The result if the English interpreting level in these seven regions will be presented in this result section, and the provincial-level result on it according to administrative division will be expatiated in the next section. Fig 4 shows the overall English interpreting level in seven regions. The size of the yellow circle embodies the regional total point. The larger the yellow circle, the higher the score. Based on Jenks natural breaks classification method [33], a data clustering method via reducing the variance within classes and maximizing the variance between classes, the level is divided into five echelons. The region of East China and Central China well-deserve the first echelon level, followed by the second echelon South China and North China. Southwest China, Northeast China and Northwest China are rated as the third, fourth and fifth echelon correspondingly. In addition, Fig 4 also overlays a background layer to show regional GDP (Gross Domestic Product), which is associated with the Discussion section. The darker the violet color, the higher the GDP level.

**Table 5:** The overall score in different geographical-level administrative regions.

Region	Overall score
East China	37.28
Central China	33.48
South China	31.50
North China	29.73
Southwest China	23.64
Northeast China	17.19
Northwest China	15.29

**Fig 5:**
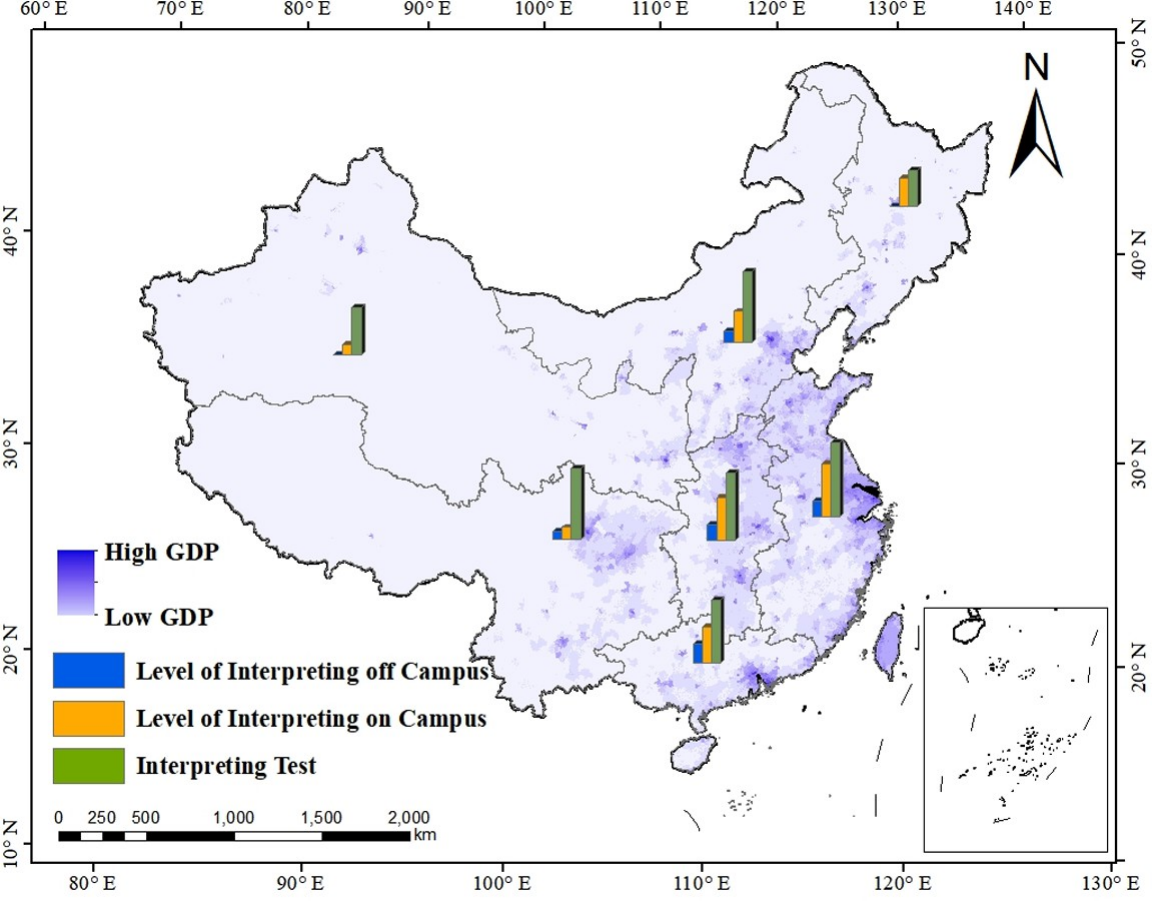
Three aspects of English interpreting level in different geographical-level administrative region.

Further analyzing three specific indicators, the situation in every region is presented in Table 6 and Fig 5. The rank of each indicator is somewhat different from the overall order mentioned above. First and foremost, the interpreting test accounts for the largest proportion of all indicators, with East China scoring top at the rank of overall regional interpreting level. However, the sequencing in the other six regions goes highly different. The interpreting test level in Southwest China orders second, stepping forward three places compared to the overall ranking, followed by North China (stepping forward one place), Central China (stepping back two places), South China (stepping back two places), Northeast China (stepping forward one place) and Northeast China (stepping back one place). Secondly, as for the level of interpreting level on campus, the ranking of it is primarily is same as that of the overall score, with East China, Central China, South China and North China obtaining the first four positions, and Northeast China, Southwest China and Northwest China the last three. Lastly, different from the previous two indicators, the score of interpreting off campus is nil in some regions such as Northeast China and Northwest China. For other regions, South China boasts the highest interpreting level outside campus, followed by Central China, East China, North China and Southwest China.

**Table 6:** The score of three aspects in different geographical-level administrative regions.

Region	Interpreting Test Score	Level of Interpreting on Campus Score	Level of Interpreting off Campus Score
East China	19.59	13.71	3.98
Central China	17.91	11.36	4.21
South China	16.83	9.60	5.07
North China	18.57	8.12	3.04
Southwest China	18.60	3.02	2.01
Northeast China	9.60	7.60	0.00
Northwest China	12.58	2.71	0.00

### 3.3 Results on English interpreting level in different provincial-level administrative regions

**Fig 6:**
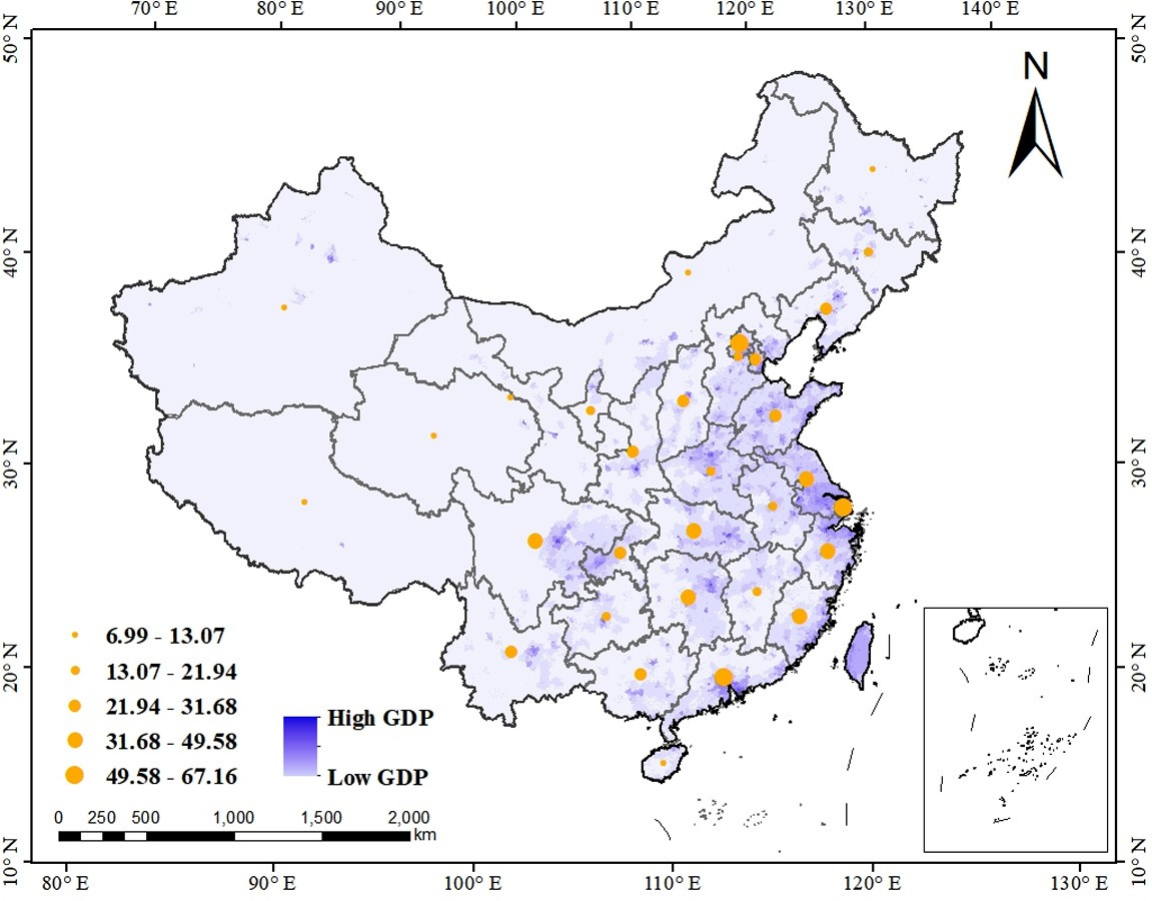
The overall English interpreting level in different provincial-level administrative region.

From the perspective of administrative division, China consists of 23 provinces, 5 autonomous regions, 4 municipalities directly under the central government and 2 special administrative regions. In this research, the regional data is collected except Taiwan, Hong Kong and Macao, thus a total of 31 groups of regional data (22 provinces, 5 autonomous regions and 4 municipalities) are included. The overall interpreting level of these 31 provincial-level administrative regions is shown as Table 7 and Fig 6. Similar to the graph of geographical-level administrative region, the size of the yellow circle represents the level of general interpreting level, which is classified into five echelons as well. On the whole, the number of the southern region in China decrease with the lower hierarchy, while that of the northern region just the reverse. There are four regions brought into the first echelon, namely Beijing, Shanghai, Guangdong and Jiangsu, in which except for Beijing, all other three regions are located in southern China. The second echelon includes sex regions, in which five (Hunan, Hubei, Fujian, Zhejiang and Sichuan) are located in the south of China, and only one (Shandong) in the north of China. As for the third intermediate echelon, eight regions are contained. Thereinto, northern (Tianjin, Shaanxi, Liaoning and Shanxi) and southern (Guangxi, Yunnan, Chongqing and Guizhou) regions are evenly balanced. Of the seven regions in the fourth echelon, the number of the northern region (Hebei, Ningxia, Henan, Jilin and Gansu) outnumber that of the southern region (Anhui, Jiangxi) for the first time. Regards to the last fifth echelon, only one region (Hainan) is located in southern China, and the other five (Xinjiang, Xizang, Heilongjiang, Nei Mongol and Qinghai) all belong to the northern part of China.

**Table 7:** The overall score in different provincial-level administrative regions.

Region	Overall Score	Ranking	Region	Overall Score	Ranking
Beijing	67.16	1	Chongqing	23.46	17
Shanghai	63.84	2	Guizhou	21.94	18
Guangdong	56.49	3	Anhui	19.26	19
Jiangsu	49.58	4	Hebei	18.96	20
Hunan	42.53	5	Ningxia	18.67	21
Hubei	40.85	6	Jiangxi	18.43	22
Fujian	40.26	7	Hainan	17.06	23
Zhejiang	37.87	8	Jilin	15.80	24
Sichuan	35.84	9	Gansu	13.07	25
Shandong	31.68	10	Xinjiang	12.49	26
Tianjin	27.54	11	Xizang	11.81	27
Guangxi	27.08	12	Heilongjiang	11.01	28
Shaanxi	25.22	13	Hainan	10.94	29
Yunnan	25.14	14	Nei Mongol	10.44	30
Liaoning	24.77	15	Qinghai	6.99	31
Shanxi	24.57	16			

**Fig 7:**
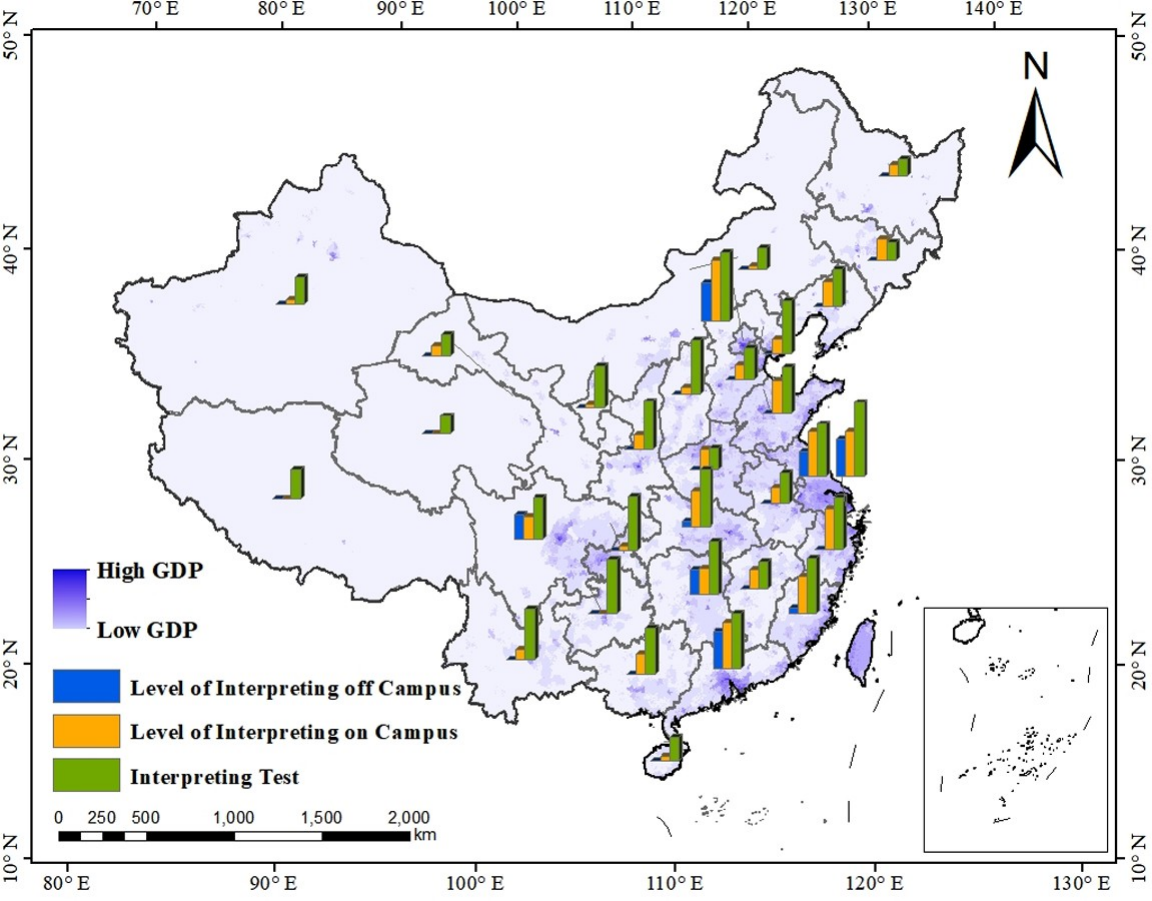
Three aspects of English interpreting level in different provincial-level administrative region.

The situation of three specific indicators is mainly the same as that of the overall interpreting level in different Chinese provincial-level administrative regions (shown as Table 8 and Fig 7). Firstly, regarding the interpreting test, of the top ten regions, seven are southern regions, with Shanghai scoring the highest in this dimension. Counting backward, the bottom of this ranking is six northern regions. Secondly, for the level of interpreting on campus, the top ten remain concentrated in the south of China, though this time the first place goes to Beijing, located northern China. It is worth noticing that the score of interpreting level on campus is naught in three regions, Guizhou, Xizang and Qinghai. Last but not least, in terms of the level of interpreting off campus, only eight southern regions in China possess the score of it, with Beijing, Shanghai and Guangdong mark in joint first place, followed by Jiangsu, Hunan, Sichuan, Fujian and Hubei.

**Table 8:** The score of three aspects in different provincial-level administrative regions.

Region	Interpreting Test Score	Level of Interpreting on Campus Score	Level of Interpreting off Campus Score
Beijing	27.51	24.44	15.21
Shanghai	30.14	18.49	15.21
Guangdong	22.41	18.87	15.21
Jiangsu	21.33	18.20	10.06
Hunan	21.57	10.90	10.06
Hubei	23.38	14.89	2.58
Fujian	22.41	15.27	2.58
Zhejiang	21.33	16.54	0.00
Sichuan	16.62	9.16	10.06
Shandong	18.43	13.25	0.00
Tianjin	21.59	5.94	0.00
Guangxi	18.81	8.28	0.00
Shaanxi	19.28	5.94	0.00
Yunnan	20.86	4.29	0.00
Liaoning	14.83	9.93	0.00
Shanxi	21.94	2.63	0.00
Chongqing	21.80	1.66	0.00
Guizhou	21.94	0.00	0.00
Anhui	12.65	6.62	0.00
Hebei	13.02	5.94	0.00
Ningxia	17.01	1.66	0.00
Jiangxi	10.83	7.60	0.00
Hainan	8.78	8.28	0.00
Jilin	7.23	8.57	0.00
Gansu	8.78	4.29	0.00
Xinjiang	10.83	1.66	0.00
Xizang	11.81	0.00	0.00
Heilongjiang	6.73	4.29	0.00
Hainan	9.28	1.66	0.00
Nei Mongol	8.78	1.66	0.00
Qinghai	6.99	0.00	0.00

To sum up, the score and ranking in Chinese different regions, both the geographical-level administrative regions and provincial-level administrative regions are arranged based on an established indicator system. The strength of the current newborn indicator system will be further discussed in the next section, coupled with analyzing the possible reason behind the ranking as well as the suggestion for future interpreting development in different regions.

## 4. Discussion

### 4.1 Strength of current regional English interpreting evaluation indicator system

The present study establishes a groundbreaking regional English interpreting evaluation indicator system with Chinese characteristics, which fills in the gap that the research on assessing regional English interpreting levels is almost blank. To construct the system, it is necessary to follow the principles of hierarchy, simplicity, comprehensiveness and operability [20, 34]. By combining qualitative and quantitative analysis, the study put forward an evaluation system containing three first-level indicators and seven second-level indicators to assess the English interpreting level in different Chinese regions. The three strengths of the current regional English interpreting evaluation indicator system are stated as follows.

Firstly, the indicator system could reflect the English interpreting level from different aspects. Through literature review and questionnaire survey on different stakeholders, indicators are ultimately determined after double screening. The level of interpreting tests, interpreting on campus and interpreting off campus represent regional interpreting levels from professional, educational and activeness perspectives. In addition, segmenting indexes like the passing rate of CATTI are set to reflect the quantity and quality of first-level indicators further. Hence, the whole indicator system abides by the rule of hierarchy and comprehensiveness. Secondly, each indicator’s weight and scoring method are validated to facilitate measurement through factor analysis and interval marking methods. The procedure improves and perfects the indicator system, and is in conformity with the principle of simplicity, thus it is user-friendly. Last but not least, the system is highly practical, since all statistics needed to be collected to reflect each indicator can be obtained from the official website or quantified from it. The objectivity of data helps improve operability, which is also one of the principles of constructing an indicator system. Therefore, this regional English interpreting indicator system meets the standard of hierarchy, simplicity, comprehensiveness and operability in the meantime.

Admittedly, deficiencies do exist in this newborn indicator system. For example, the concept of quality of interpreting level off campus is relatively complex, which is not only represented by teaching resources of interpreting training institutions. Considering the non-availability of some data, the present study only selects teaching resource as its second-level indicator. Hence, the indicator system hope can be optimized and upgraded in future research work.

### 4.2 Uneven national English interpreting level and reasons behind

Based on the results produced in the previous section, the English interpreting level is uneven and unbalanced across the country in general. Regardless of results on geographical-level or provincial-level administrative regions, it shows that the level of English interpreting is higher in the southern and eastern parts of China compared to that of northern and western China. The reason behind it can be roughly divided into three categories: geography, economy and education.

**Fig 8:**
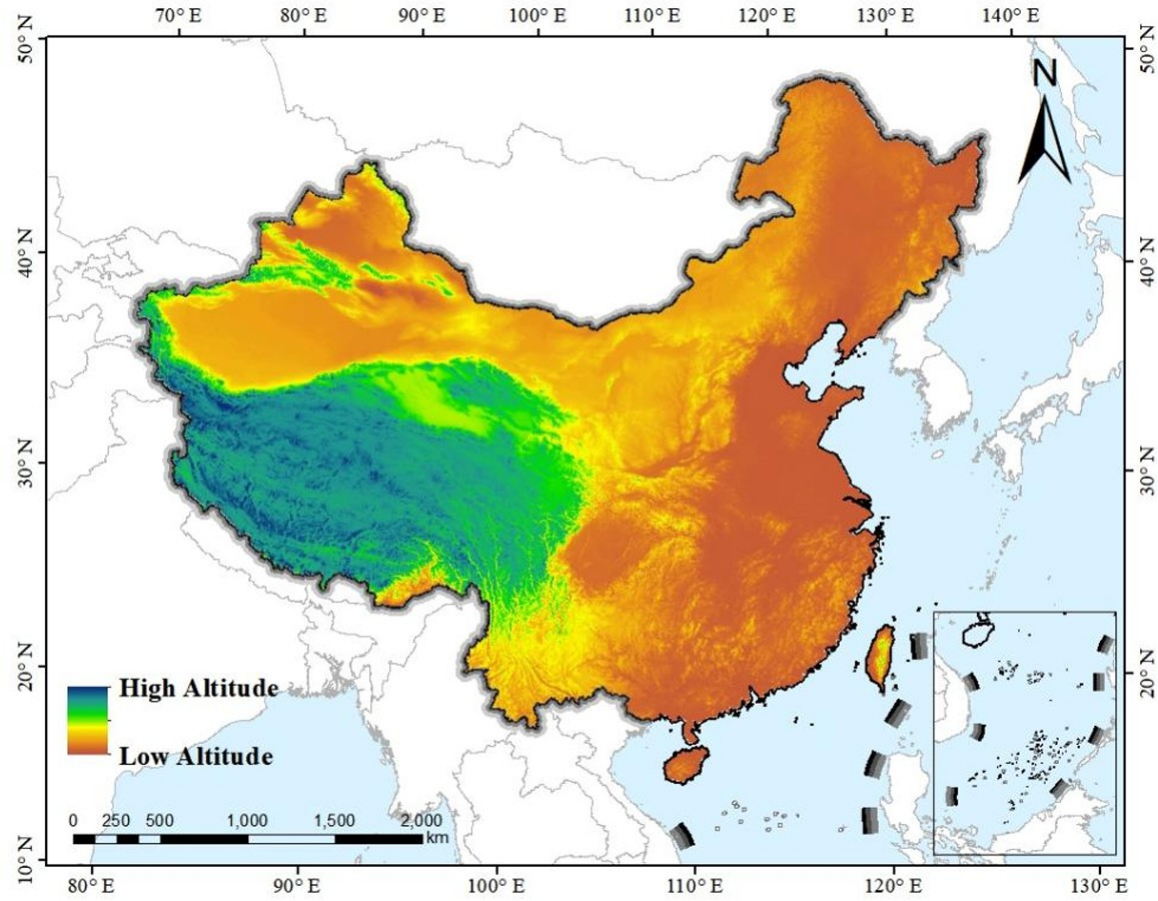
Topographic map of China.

Geographically speaking, the terrain of China is high in the west and low in the east, presenting a ladder-like distribution [35]. According to the altitude, the terrain can be divided into three levels: first-level terrain with an average altitude of above 4500 meters in the west of China, second-level terrain with an average altitude between 1000 and 2000 meters in the middle part of China and third-level terrain with an average altitude of below 500 meters in the east of China (shown as Fig 8). The third-level terrain extends to the ocean naturally, which provides an unique advantage for it to international trade and commerce. Due to geographical advantage, international exchanges and foreign trade are heavily concentrated in coastal regions [36], which promotes the appliance of English interpreting to a very large extent. The geographical reason can also explain the ranking of regional English interpreting level in this research. Among the top three (East China, Central China and South China), two of them are coastal areas (East China and South China), presenting a more advanced level in these regions.

The economy is also very likely to be the reason leading to the current uneven situation. The ranking of the current study maintains high consistency with regional GDP order. For example, the region of four first-tier cities retains the leading position in both national GDP and English interpreting level ranking. In addition, the result is also closely linked with the proportion of the tertiary industry, an index representing the degree of economic development [37]. The classification standard divided the primary, secondary and tertiary industry is varies from country to country. According to the division criteria of China, the tertiary industry is the service industry [38], which refers to other industries other than the primary industry (e.g. agriculture, forestry, animal husbandry and fishery) and the secondary industry (e.g. mining, manufacturing, electricity, etc). With regards to interpreting, no matter whether interpreting teaching or interpreting service, they all belong to the tertiary industry. In consequence, it is understandable that the region, such as Beijing and Shanghai, with higher tertiary industry proportion possess more developed English interpreting levels.

Apart from the geographical and economic reasons aforementioned, education, higher education in particular, is also correlated with the current ranking in all likelihood. According to the ranking of Chinese University Translation and Interpreting Ability Index released by the Research Centre for Country-specific Translation and Interpretation Capacity (RECTIC) attached to Beijing Foreign Studies University, of the top 20 universities, 17 are located in the eastern part of China [28]. It indicates that there is a prominent gap in professional training at the university level between the east and west of China. Summing up the above, it is rational to conclude that geographical, economic and educational factors are highly possible to be the reason for the imbalanced regional English interpreting level in China.

### 4.3 Suggestions on sustainable development of interpreting in different regions

Given uneven English interpreting levels across the country, several suggestions are propounded to remedy this issue for future sustainable development. First of all, for those regions without interpreting major in colleges or universities, it is necessary for them to set up professional interpreting programs for both under and postgraduate students. This initiative helps cultivate and reserve interpreting talents, laying a solid foundation for sustainable development in the future.

Secondly, the development of the interpreting level could take advantage of an opportunity for Chinese urban agglomeration development. Facing the imbalance reality, the Chinese government has established a new model with central cities leading the development of urban agglomerations and urban agglomerations driving regional development, so as to promote the integrated and interactive development of regional sectors [39]. Taking the Yangtze River Delta Urban Agglomerations for example, it is an important engine for economic development, and one of the regions with the best urbanization foundation in China, including Shanghai, Jiangsu province, Zhejiang province and Anhui province [40, 41]. In the current interpreting ranking, Shanghai and Jiangsu are rated in the first echelon, Zhejiang province the second and Anhui province the fourth. Thus, the former three regions could offer assistance to Anhui province, narrowing the disparity within urban agglomeration. This action is particularly beneficial for improving the interpreting level off campus, since the free interpreting market is the most flexible and can respond in the first time.

The third suggestion is proposed for remote areas with lower interpreting rankings. It is practical for these regions to promote interpreting development integrating local characteristics. For instance, Xinjiang and Xizang are both located in the border area with abundant tourism resources, but are ordered in the fifth echelon of the interpreting level. Therefore, the local government could combine interpreting with tourism resources, carrying forward local traditional culture through the power of interpreting.

Last but not least, the development of regional interpreting should hitch a ride of economic development. On account of the fact that the result visually shows that the English interpreting aptitude across China echos with regional economic performance, the interconnectedness between regional interpreting level and regional economy is self-evident, which provides a god-given opportunity for region such as Hainan (ranking 29 of 31 in the present study). In 2018, the Chinese government supports the launch of the China (Hainan) Pilot Free Trade Zone [42]. According to Gile’s viewpoint, trading and exchanging goods, being the primary mean of establishing links between communities using different languages, give rise to interpreting development [43]. Hopefully, regions in such conditions could seize this opportunity and catch up from behind. In short, all suggestions only point to one purpose, realizing the balance and sustainable development of English interpreting in China.

## 5. Conclusions

### 5.1 Policy implications

Based on the above results and discussion, some specific suggestions are put forward to the government, policymakers and implementers. First, the central government should further implement the decentralization policy, giving local governments more autonomy to develop their own English and interpreting education system. Provincial governments in northeast and northwest China should pay closer attention to cultivating student English interpreting ability. Second, for policymakers, they should strengthen links with university administrators and related institution managers for in-depth understanding of their difficulties and needs. Third, implementers including teachers also play a central role in the interpreting industry. As the main force in cultivating interpreting talents, they should actively report their teaching situation to administrators, so a more flexible and appropriate plan can be built from top to bottom.

### 5.2 Limitations and future studies

Admittedly, there are some limitations in the current study. Firstly, although the selected indicators were finally determined based on two steps—literature review and questionnaire survey, some factors that could also reflect regional interpreting level such as the economic effect brought by interpreting may be ignored in this study. Hence, in the future, this new-born indicator system can be further improved to a more comprehensive and inclusive one. Secondly, only data from the year 2021 were collected and input in the indicator system, which is less convincing than acquiring data within the recent five years. Therefore, it is suitable for future researchers to conduct a tracking study. Lastly, the study only covered areas in mainland China, so the data from Hong Kong, Taiwan and Macao can be supplemented in future studies.

To sum up, for the sake of sustainable interpreting development, an indicator system for regional English interpreting level was established using the methods of literature review, questionnaire survey, factor analysis and interval marking, which includes 2 layers, 3 first-level indicators and 7 second-level indicators. The overall and first-level indicators’ scores are ranked among 7 geographical-level administrative regions and 31 provincial-level administrative regions. The results showed that the English interpreting level across China is uneven: the level of English interpreting is higher in the southern and eastern parts of China compared to that of northern and western China. Based on the indicator system and rating score, possible reasons, geographical, economic and educational factors behind it are analyzed, and targeted suggestions are put forward for future English interpreting balanced and sustainable development. The innovative indicator system and findings provide an effective reference for further regional English interpreting level study to increase the sustainable capability of regional comprehensive development.

## Supporting information

S1 Appendix (DOCX)
