# Supplementary material for: Regional gap and sustainable development of interpreting level in mainland China: A statistics and GIS-based study
Source: PLoS One. 2024 Mar 29;19(3):e0295505. doi: 10.1371/journal.pone.0295505 (PMC10980182; doi:10.1371/journal.pone.0295505)
Supplement: S1 Appendix — (DOCX) [file pone.0295505.s001.docx]

**Appendix**

Indicator Selection Questionnaire for Regional English Interpreting Level

Here are a number of questions related to indicator of assessing regional English interpreting level. Please tick the option to indicate the extent to which you agree or disagree with that statement. Please respond with the answer that most corresponds to your real thoughts, as there are no right or wrong answers.

| Disagree  strongly | Disagree  a little | Neither agree  nor disagree | Agree  a little | Agree  Strongly |
| --- | --- | --- | --- | --- |
| 1 | 2 | 3 | 4 | 5 |

1. I think interpreting test can be chose as an indicator to assess regional English interpreting level.

Disagree strongly (1)

Disagree a little (2)

Neither agree or disagree (3)

Agree a little (4)

Agree strongly (5)

2. I think the number of interpreting test type can be chose as an indicator to assess interpreting test.

Disagree strongly (1)

Disagree a little (2)

Neither agree or disagree (3)

Agree a little (4)

Agree strongly (5)

3.I think passing rate of CATTI can be chose as an indicator to assess interpreting test.

Disagree strongly (1)

Disagree a little (2)

Neither agree or disagree (3)

Agree a little (4)

Agree strongly (5)

4.I think the level of interpreting on campus can be chose as an indicator to assess regional English interpreting level.

Disagree strongly (1)

Disagree a little (2)

Neither agree or disagree (3)

Agree a little (4)

Agree strongly (5)

5.I think the number of colleges and universities with interpreting major can be chose as an indicator to assess the level of interpreting on campus.

Disagree strongly (1)

Disagree a little (2)

Neither agree or disagree (3)

Agree a little (4)

Agree strongly (5)

6.I think interpreting teachers’ scientific research level can be chose as an indicator to assess the level of interpreting on campus.

Disagree strongly (1)

Disagree a little (2)

Neither agree or disagree (3)

Agree a little (4)

Agree strongly (5)

7.I think students’ performance in interpreting contest can be chose as an indicator to assess the level of interpreting on campus.

Disagree strongly (1)

Disagree a little (2)

Neither agree or disagree (3)

Agree a little (4)

Agree strongly (5)

8.I think the level of interpreting off campus can be chose as an indicator to assess regional English interpreting level.

Disagree strongly (1)

Disagree a little (2)

Neither agree or disagree (3)

Agree a little (4)

Agree strongly (5)

9.I think the number of interpreting training institution can be chose as an indicator to assess the level of interpreting off campus.

Disagree strongly (1)

Disagree a little (2)

Neither agree or disagree (3)

Agree a little (4)

Agree strongly (5)

10.I think teaching resource can be chose as an indicator to assess the level of interpreting off campus.

Disagree strongly (1)

Disagree a little (2)

Neither agree or disagree (3)

Agree a little (4)

Agree strongly (5)

11.I am a/an______

Interpreting discipline expert

Professional interpreter

Student interpreter

From your perspective, whether there are some other indicators can put into the system to assess regional English interpreting level? Your comments are welcome.
